# Evaluating the feasibility and acceptability of an adapted fencing intervention in breast cancer surgery post-operative care: the RIPOSTE pilot randomized trial

**DOI:** 10.3389/fonc.2024.1335442

**Published:** 2024-04-11

**Authors:** Sabrine Hasnaoui, Aurélie Van Hoye, Marc Soudant, Christine Rotonda, Andréia Carvalho de Freitas, Didier Peiffert, Cécile Delattre, Julien Raft, Margaux Temperelli, Edem Allado, Oriane Hily, Bruno Chenuel, Dominique Hornus-Dragne, Abdou Y. Omorou, Mathias Poussel

**Affiliations:** ^1^ Université de Lorraine, INSERM, UMR 1319, INSPIIRE, Nancy, France; ^2^ University of Limerick, Physical Activity for Health Research Center, Limerick, Ireland; ^3^ Université de Lorraine, CHRU-Nancy, Inserm CIC-1433 Clinical Epidemiology, Nancy, France; ^4^ Université de Lorraine, INSERM, UMR 1319, INSPIIRE, Metz, France; ^5^ Université de Lorraine, Centre Pierre Janet, Metz, France; ^6^ Lorraine Institute of Oncology, Department of Radiation Oncology, Vandoeuvre-Lès-Nancy, France; ^7^ Lorraine Institute of Oncology, Supportive Care Unit, Vandoeuvre-Lès-Nancy, France; ^8^ Lorraine Institute of Oncology, Department of Anesthesiology, Vandoeuvre-Lès-Nancy, France; ^9^ Université de Lorraine, INSERM UMR-S 1116 Equipe 2, Nancy, France; ^10^ Université de Lorraine, CHRU-Nancy, University Centre of Sports Medicine and Adapted Physical Activity, Nancy, France; ^11^ Université de Lorraine, DevAH, Department of Physiology, Nancy, France; ^12^ Solution RIPOSTE, Balma, France; ^13^ The French National Platform Quality of Life and Cancer, Nancy, France

**Keywords:** breast cancer, adapted physical activity, fencing, quality of life, fatigue

## Abstract

**Background:**

Adapted physical activity programs have shown promising results in reducing the physical, social and psychological side effects associated with breast cancer, but the extent to which they can be effectively adopted, implemented and maintained is unclear. The aim of this study is to use the framework to guide the planning and evaluation of programs according to the 5 following keys: Reach, Effectiveness, Adoption, Implementation and Maintenance (RE-AIM) framework to evaluate a fencing program under the French acronym RIPOSTE (Reconstruction, Image de soi, Posture, Oncologie, Santé, Thérapie, Escrime) literally in English (Reconstruction, Self-Image, Posture, Oncology, Health, Therapy, Fencing). This program is an innovative intervention focused on improving the quality of life (QoL) of breast cancer surgery patients through fencing.

**Methods:**

A convergent mixed methods pilot study was conducted to preliminary evaluate the different RE-AIM dimension of the pilot program. Twenty-four participants who have just undergone surgery for invasive breast cancer were randomly allocated in two groups: one group started immediately after their inclusion (Early RIPOSTE group) and the other started 3 months following their inclusion (Delayed RIPOSTE group). Participants answered a questionnaire at inclusion and at the end of the program on QoL, shoulder functional capacity, fatigue, anxiety-depression and physical activity.

**Results:**

RIPOSTE program was able to reach mainly young and dynamic participants, attracted by the originality of fencing and keen to improve their physical condition. Regarding effectiveness, our results suggest a trend to the improvement of QoL, shoulder functional capacity, fatigue and anxiety-depression state, even without any significant differences between the Early RIPOSTE group and the Delayed RIPOSTE group.

**Discussions:**

The cooperation, exchanges and cohesion within the group greatly facilitated the adoption of the program, whereas interruptions during school vacations were the main barriers. The intervention was moderately well implemented and adherence to the protocol was suitable.

**Conclusion:**

RIPOSTE is an acceptable and effective program for involving breast cancer survivors in physical activity, that needs to be tested at a larger scale to investigate its effectiveness, but has the potential to be transferred and scaled up worldwide.

## 1 Introduction

Breast cancer (BC) remains the most diagnosed cancer and the leading cause of cancer death among women in France (1). Treatment improvements have enhanced cancer survivorship, leading to a 5-year survival rate of 89% for people diagnosed with BC (1). These high cancer survival rates have led to increased exposure to physiological (e.g., fatigue, mobility problems) and psychological (e.g., anxiety, depression, low quality of life) adverse side effects associated with the disease and related common treatments (2).

One of the key non-pharmacological treatments showing promising results in managing these adverse effects are physical activity (PA) programs, implemented at each stage of the cancer progression. Indeed, PA interventions have been shown to enhance quality of life (QoL), reduce symptoms and simultaneously minimize the risk of recurrence and comorbidities among patients living with and beyond cancer (3–9). Based on these evidence-based benefits, a fencing program under the French acronym RIPOSTE (Reconstruction, Image de soi, Posture, Oncologie, Santé, Thérapie, Escrime) literally in English (Reconstruction, Self-Image, Posture, Oncology, Health, Therapy, Fencing) was created for BC surgery patients in 2014 (10). This adapted physical activity (APA) program has been considered by public health and medical stakeholders as an innovative intervention through fencing (10, 11). APA is defined as “a cross-disciplinary body of practical and theoretical knowledge directed toward impairments, activity limitations, and participation restrictions in physical activity” (12). The relevance of fencing as an APA program in the context of BC was suggested quite naturally, as it is well adapted to combating fatigue, pain and reduced arm mobility, as well as improving patients’ physical and psychological well-being (10, 13, 14). More specifically, the use of the saber (and not the foil or epee) allows maximum mobilization and opening of the shoulder, due to the particularity of this weapon. The RIPOSTE program will celebrate its 10th anniversary in 2024. Currently, over 100 fencing clubs offer this adapted fencing program in France and a few more abroad. The aim of the scientific committee is to further develop this program for as many clubs as possible, always with the same high-quality standards, and to spread more widely internationally. Contacts have already been established with the International Fencing Federation (IFF) and with some North American clubs, so that adapted fencing could be considered a valid non-pharmacological treatment worldwide. But more data on its benefits and implementation process is needed to anticipate this process (11, 15).

To date, despite large evidence on the benefits of PA for people with BC, the extent to which PA programs could be effectively adopted, implemented and sustained remains unclear (16–20). Previous literature has shown mixed results over the long-term impact of interventions on PA practice (16, 17). Indeed, several programs have achieved limited success in improving PA among adults, particularly due to a lack of maintenance and sustainability of practice once the intervention ends (18, 19). Also, the existing evidence has focused almost exclusively on the high internal validity – or the magnitude of effect as a key indicator for programs impact. However, issues related to external validity - how the results of a study can be generalized to other contexts (21) - received less attention (20). Assessing external validity when synthesizing the results of this type of program is important to support sustainably their translation into practice (22–24). An accurate and comprehensive evaluation of the system supporting the implementation process is needed in order to understand what works, for whom and how (25, 26).

While RIPOSTE program has provided some success on the field across France, its evaluation in terms of effectiveness and implementation process has remained at its infancy, with no proper evaluation so far. Determining the extent to which this program is effective, acceptable and implementable is a necessary preliminary step. These findings would help to enrich the literature on how to best intervene to enhance treatment and recovery among BC patients, considering both patient and professional preferences and adoption, to facilitate transfer from a research program to a field daily practice. The aim of this preliminary study was to evaluate the RIPOSTE program effectiveness and implementation process.

## 2 Method

### 2.1 Study design

A convergent mixed methods (QUAN+QUAL) pilot study was conducted using the framework to guide the planning and evaluation of programs according to the 5 following keys: Reach, Effectiveness, Adoption, Implementation and Maintenance (RE-AIM) framework as a guide to evaluate RIPOSTE (27). Quantitative and qualitative data were collected at the same time and combined during data analysis in a convergent design, giving the same weight to each type of data. A commonly used framework to assess research implementation is the RE-AIM framework which conceptualizes the public health impact of interventions as a function of the 5 dimensions of RE-AIM (**Table 1**) (28). This framework extends beyond standard measures of efficacy, considering individual and organizational factors that can either facilitate or impede the successful implementation and dissemination of a program (29, 30). A full description of the design and methodological protocol of the program have been reported previously (10). This research complies with the Helsinki declaration and received ethical authorization by the French committee for individual protection (CPP Sud Méditerranée IV, N°ID-RCB: 2020-A01916-33). This research is registered on clinicaltrials.gov (NCT04627714).

**Table 1:** RE-AIM components in the context of the RIPOSTE trial.

Dimension	Definition applied for this study	Outcome measures	Data sources
**Reach**	The proportion and representativeness of eligible individuals who participate in the program	- Absolute number of participants - Proportion of participants in the target population - Characteristics of participants compared to non-participants - Barriers and facilitators to participation according to participants, fencing masters and sports physicians	- Clinical examination - Interviews
**Effectiveness**	The effects of the program on relevant outcomes *including behavioral and QoL outcomes*	- Post-surgical effectiveness of fencing versus no fencing on 3-month QoL outcomes - Post-surgical effectiveness of fencing versus no fencing on 3-month changes in global shoulder functional capacity, fatigue, anxiety-depression and PA - Measure of inter-individual differences related to individual characteristics	- Clinical examination - QLQ-C30 - QLQ-BR23 - DASH - MFI-20 - HADS
**Adoption**	The proportion and representativeness of patients that adopt the program	- Participation and withdrawal rates - Number of sessions attended out of total number of sessions (per participant) - Barriers and facilitators to patients uptaking the program according to participants, fencing masters and doctors	- Interviews
**Implementation**	The fidelity of the program to the various elements of its key functions and components	- Consistency of implementation (across individuals/time/settings) - Adaptations made to intervention during study - Participant and professional perceptions of the barriers and facilitators to implementation	- Interviews
**Maintenance**	Long-term effects of the program on behavior maintenance	- Continuation of RIPOSTE or PA by participants - Program perspectives - Prospects for change or improvement in future implementation (according to sports physicians, fencing masters and researchers)	- Interviews

### 2.2 Participants and recruitment

**Figure 1:**
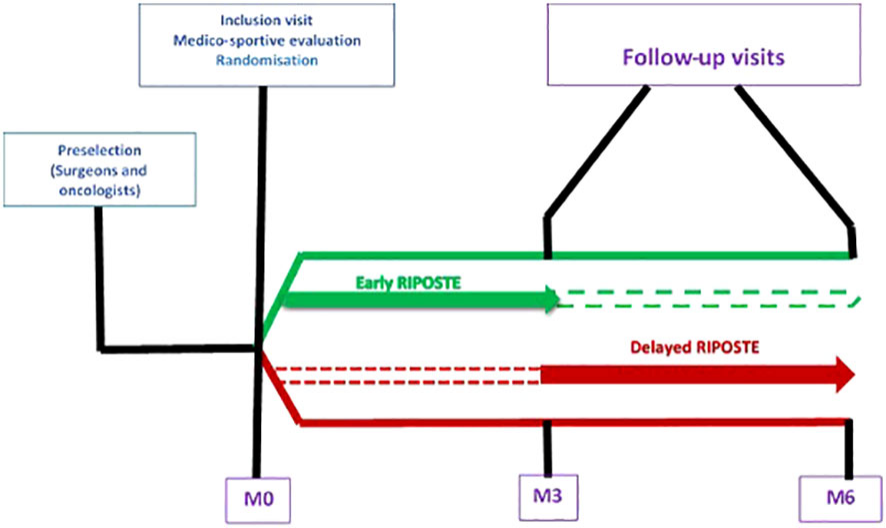
Study design and patient allocation.

From November 10^th^ to January 19^th^ 2021, patients diagnosed with invasive BC were screened from 3 centers in Northeastern France (University Hospital of Nancy CHRU, France), Institut de Cancérologie de Lorraine, Nancy, France and Hôpital d’Instruction des Armées Legouest, Metz, France). Among the 309 patients screened, 24 met the inclusion criteria (10) and were selected from those who had recently undergone breast surgery for invasive BC. The surgeon, anesthesiologist or oncologist referred them to a sports physician within 2 to 4 weeks after surgery for an inclusion visit. All participants were provided with an information letter outlining the research and completed written informed consent. Patients were then randomized, with centralized 1:1 computerized requested via the web interface (e-crf) by the investigator with a sequential minimization of factor 85%, to one of two groups. The Early RIPOSTE group received one fencing session (duration of 1–1.5 h/session) per week from inclusion and for 3 months in the program. The Delayed RIPOSTE group received a follow-up without fencing for the first 3 months after inclusion and then the program (i.e. one fencing session per week) for the last 3 months (see **Figure 1** for sample allocation and intervention description). Recruitment occurred between November 2021 and January 2022. They followed fencing sessions that had a common structure in the fencing French halls in the cities of Vandoeuvre-lès-Nancy and Thionville. Participation in all sessions was not required to be considered a study participant, but the level of participation was analyzed as a reach indicator.

### 2.3 Data collection

Included patients underwent a sports medical examination at inclusion, 3 months after randomization, and 6 months after randomization. Quantitative data regarding each RE-AIM dimension were collected, without blinding of the randomization arm, at each time. Qualitative interviews were also conducted with 10 patients (5 patients per group), 3 sports medicine physicians, and 1 fencing master at the end of follow-up. Methods of data collection included clinical examination, questionnaires and interviews.

#### 2.3.1 Quantitative outcomes

##### 2.3.1.1 Clinical examination

The sociodemographic and clinical factors of study patients at enrollment included age, marital and familial status, number and age of children, education level, current employment status, socio-professional category, social support, sleep habits and PA. At inclusion, data on disease stage, age of diagnosis, age and type of breast surgery were also collected. Medical data such as additional treatments, weight gain, presence of lymphedema and comorbidities were collected at the baseline, 3-month and 6-month follow-up by the sports physician or the clinical study technician. At each visit, the sports physician also collected the shoulder functional score from the Constant index as well as injuries or any other pathological symptoms related to fencing.

##### 2.3.1.2 Questionnaires

At the end of each visit, a notebook was given to the patient to answer the different questionnaires. The evolution of QoL at 3 months was measured by the third version of the European Organization for Research and Treatment of Cancer (EORTC) Quality of Life Questionnaire Core 30 (QLQ-C30) (31) and BC specific Quality of Life Questionnaire module (QLQ-BR23) (32). The QLQ-BR23 is a supplementary 23-item questionnaire module that measures BC symptoms as well as treatment side effects.

Global functional capacity of both upper extremities was assessed using the Disabilities of the Arm, Shoulder and Hand (DASH) measurement, validated in French (33).

Fatigue over the previous days was measured by the Multidimensional Fatigue Inventory (MFI-20), validated in French (34).

The 14-item Hospital Anxiety and Depression Scale (HADS) was used to measure a standardized anxiety and depression score (35).

PA was assessed by the International Physical Activity Questionnaire (IPAQ), a validated international questionnaire that has been translated into several languages including French (36).

#### 2.3.2 Qualitative interviews

Semi-structured retrospective narrative interviews were conducted with 10 participants (5 per group) within weeks of the 3-month follow-up data collection period. These interviews ranged from 40 to 60 minutes in duration and consisted of asking participants open-ended questions pertaining to their experiences with the program, identified difficulties and facilitators, and their perceptions of how the program could be improved. Semi-structured interviews were also conducted with 3 sports physicians (SP1, SP2, SP3) and 1 fencing master (FM) at the end of the follow-up to identify indicators of efficacy, including patient acceptability, from the professionals’ perspective. Interviews were recorded and transcribed verbatim.

### 2.4 Data analysis

#### 2.4.1 Quantitative data

The change in QLQ C30 global health status score at 3 months was analyzed using Student’s t test, after checking normality and homoscedasticity assumptions, between the randomization arms according to intention to treat principle. This analysis was done on complete cases. Multivariate mixed modeling was also performed with patient random effect in order to take into account repeated measures correlations. For sensitivity analysis purposes, the same analysis was realized using multiple imputations. Patterns of missing data were studied and multiple imputations were used when observations with at least one missing data exceeded 5%, the von Hippel method was used to determine the number of imputation datasets. Other comparisons used the same non-adjusted statistical methods for the other measured variables replacing Student’s ‘t tests’ by Welch’s tests and Wilcoxon-Mann Whitney tests when homoscedasticity and normality, respectively, were not satisfied. Considering the second period (M6), without covariates adjustment, a two-stage crossover model using Grizzle model based on Student’s ‘t tests’ evaluating the carryover (residual of the intervention), direct of the intervention and period effects. Confounding effects were taken into account in the second time with a mixed model explaining each score progression since baseline with the same selection strategy as the mixed model restricted to the first 3 months. The data analysis was generated using SAS/STAT software, Version 9.4 of the SAS System for Microsoft Windows.

#### 2.4.2 Qualitative data

Patient and professional discourse were fully transcribed and cross-referenced. A deductive thematic content analysis (37), based on each RE-AIM category, was conducted using Dedoose (Version *9.0.17*, cloud application for managing, analyzing, and presenting qualitative and mixed method research data (2021). Los Angeles, CA, USA). The coded data were reviewed and compared within each dimension to identify common as well as contrasting ideas and experiences by the first author and cross-checked by a second author. Descriptive summaries for each category along with illustrative quotes were developed from these data and discussed within the team.

## 3 Results

In order to best reflect the aims of the study, the results are presented under the RE-AIM framework.

### 3.1 Reach

#### 3.1.1 Number and proportion of participants

Among the 309 patients screened, 73 were eligible for enrollment. Among them, 49 were not included (refusal) so 24 patients were allocated either in the Early RIPOSTE group (n = 11) or the Delayed RIPOSTE group (n = 13).

#### 3.1.2 Characteristics of participants

Sociodemographic and clinical characteristics of the study sample are presented in **Table 2**.

**Table 2:** Sociodemographic and clinical characteristics of the study sample.

	Early RIPOSTE		Delayed RIPOSTE		Total		stdiff^†^
	N=11		N=13		N=24		
Age							0.094
Mean (SD)	58.8 (10.84)		57.7 (13.03)		58.2 (11.83)	
Family situation							
Living alone	3	(27.3%)	4	(30.8%)	7	(29.2%)	0.077
Living with:	8	(72.7%)	9	(69.2%)	17	(70.8%)	
Spouse/Partner	7	(63.6%)	9	(69.2%)	16	(66.7%)	0.119
Children	0	(0.0%)	1	(7.7%)	1	(4.2%)	0.408
Parents	0	(0.0%)	0	(0.0%)	0	(0.0%)	*N/A*
Other	1	(9.1%)	0	(0.0%)	1	(4.2%)	0.447
Marital status							0.668
* NR*	1		0		1	
Married	5	(45.5%)	7	(53.8%)	12	(50.0%)
Civil union partner	2	(18.2%)	2	(15.4%)	4	(16.7%)
Single	1	(9.1%)	1	(7.7%)	2	(8.3%)
Separated	1	(9.1%)	0	(0.0%)	1	(4.2%)
Divorced	1	(9.1%)	2	(15.4%)	3	(12.5%)
Widowed	0	(0.0%)	1	(7.7%)	1	(4.2%)
Number of children							0.487
Mean (SD)	2.0 (1.95)		1.2 (1.09)		1.6 (1.56)	
Socio-professional category							0.666
Farmers	0	(0.0%)	0	(0.0%)	0	(0.0%)
Craftsman, shopkeeper and business owner	1	(9.1%)	1	(7.7%)	2	(8.3%)
Executives and higher intellectual professions	4	(36.4%)	5	(38.5%)	9	(37.5%)
Intermediate profession	0	(0.0%)	1	(7.7%)	1	(4.2%)
Employees	4	(36.4%)	4	(30.8%)	8	(33.3%)
Workers	0	(0.0%)	0	(0.0%)	0	(0.0%)
Retired	1	(9.1%)	2	(15.4%)	3	(12.5%)
Without Professional activity	1	(9.1%)	0	(0.0%)	1	(4.2%)
Having a caregiver							0.408
* NR*	1		0		1	
No	10	(90.9%)	12	(92.3%)	22	(91.7%)
Yes	0	(0.0%)	1	(7.7%)	1	(4.2%)
Number of days since diagnostic							0.157
Mean (SD)	114.8 (91.71)		131.3 (116.30)		123.8 (103.85)	
Disease stage							0.570
Stage 1	3	(27.3%)	2	(15.4%)	5	(20.8%)
Stage 2	5	(45.5%)	8	(61.5%)	13	(54.2%)
Stage 3	3	(27.3%)	2	(15.4%)	5	(20.8%)
Stage 4	0	(0.0%)	1	(7.7%)	1	(4.2%)
Number of days since breast surgery							0.082
Mean (SD)	18.5 (7.58)		19.0 (5.58)		18.8 (6.43)	
Type of treatment							
** Mastectomy**	9	(81.8%)	11	(84.6%)	20	(83.3%)	0.075
** Tumorectomy**	8	(72.7%)	9	(69.2%)	17	(70.8%)	0.077
Radiotherapy							0.194
* NR*	1		0		1	
No	6	(54.5%)	9	(69.2%)	15	(62.5%)
Yes	4	(36.4%)	4	(30.8%)	8	(33.3%)
Chemotherapy							0.354
* NR*	1		0		1	
No	7	(63.6%)	11	(84.6%)	18	(75.0%)
Yes	3	(27.3%)	2	(15.4%)	5	(20.8%)

†Standardized difference (stdiff) = effect size (Yang, D., & Dalton, J. E. (2012). A unified approach to measuring the effect size between two groups using SAS^®^. 6).

#### 3.1.3 Profile and reasons for participation

Participant’s quotations in regard to their participation and experience are described in **Table 3**. Elderly or sedentary patients were less likely to participate, or the sports medicine physician did not suggest the program to them. Most of the participants were dynamic and physically active prior to their cancer. The main reasons for participation were the originality of fencing, the symbolism of “combat”, and the fact that the program was free, as well as known to sports medicine physicians. Their participation was also motivated by a desire to take control over their lives and distance themselves from the cancer environment. At the same time, most patients saw the program as an opportunity to improve disease-related symptoms and manage the side effects of treatment. A few were more interested in taking part in a study and contributing to cancer research. While some participants expressed a desire to share an activity with other cancer survivors, the more introverted ones were held back by the collective dimension of fencing. From an organizational point of view, the distance between home and the fencing gym, the day and time suited most of the participants, principally because they were no longer working and therefore had few constraints. However, those with children regretted that the training sessions were not offered during school hours.

**Table 3:** Participant’s quotation in regard to their participation and experience to RIPOSTE.

	Category	Quotes
Reasons for participation	Dynamic and active profiles	*« There are probably people for whom it didn’t even occur to me to suggest it because they didn’t have the right profile. They may be ladies who are too old, or not at all dynamic. (…) It’s an almost unconscious selection. (…) I’m sure there are people who missed out, I didn’t systematically suggest it to everyone. » (SP2)* *« The problem with all these adapted physical activity programs is that the people who respond to them are people who are convinced that physical activity is important, and in the end they are people who were already somewhat athletic before or who already practiced physical activities. (…) The profile of the patients is rather young and dynamic. » (SP2)*
	Originality of fencing	*« It’s an original and little-known sports program, and one that arouses the curiosity of patients. » (SP1)*
	Symbolism of « combat » associated with fencing	*« That is also a fight against us, against what happens to us. So in the fact of having a saber and doing fencing, there is also the symbolism “we defend ourselves”. On a psychological level, I find it good to have this approach (…) of someone who defends herself. »* (P1)
	Good knowledge of the program among sports physicians	*« I took the time to meet the fencing master and visit the weapons room. (…) Having been there and seen things with my own eyes, and having done a year of fencing myself, (…) I think that made it easier for me. (…) I knew what it was and where I was sending my patients. » (SP2)* *« We know what we’re really talking about, we know which programs we’re sending our patients to. I think that’s essential. You can never explain things as well as when you know what you’re talking about. (…) Because if you’re just giving a succinct presentation, telling them they can do fencing and that’s what’s on offer, it’s not the same as if you’re really convinced that it’s a great activity and that it won’t be harmful for them because we’ve tried it ourselves. And I think that’s obviously not the same way of presenting things. » (SP2)*
	Getting out of the cancer environment	*« It was going to take me out of the context of the hospital, the treatments.»* (P2)
	Improvement of symptoms and side effects	*« I thought it would be a good rehabilitation (…) for my breast, for my arm. »* (P7)
	Improvement of general well-being	*« It is the fact of wanting to stay in shape, in good health, to age well, to heal better perhaps. »* (P1)
	Collective dimension	*« Curious (…) to share. Because I told them it was with other patients who had the same pathologies. So they were curious to meet other people too, to be able to talk, to take their minds off things. » (SP1)*
	Participation in cancer research	*« For others. Honestly, that’s what made me want to do it. Because if I had wanted to do sports for myself, I would have gone to the pool or done my own sports in my corner actually. So I go back to giving a little something to other patients, to research, to studies. »* (P4)
	Day and time	***«*** *Friday morning was good. (…) The schedules were good. Most of us haven’t gone back to work yet, so it was good. »* (P10)
	Distance between home and the fencing gym	*« I didn’t live far from Nancy. (…) Distance played a role. »* (P9)
	Free access	*« There is the fact that it is free. (…) And that’s good because otherwise it’s a barrier. Because, honestly, for three months, I wouldn’t have paid. »* (P8) *« Fencing isn’t the cheapest sport in the world, let’s face it. That’s why we wanted the program to be free for patients, at least for the first year. » (SP3)*
Reasons for non-participation	Collective dimension	*« It can be a bonding experience for those who need it most (…) I’m pretty self-sufficient and lonely, so I prefer activities I can do alone. »* (P6) *« Some people find it very convenient to get together with others who are going through the same thing. And there are people who, on the contrary, don’t feel like it. (…) So it’s a question of choice. (…) If it’s not their character, if they’re more introverted and don’t want to talk to other people, it’s not for them. I think one of the essential things for a physical activity to work is for the patient to adhere to it and enjoy it. If it goes against what they like to do, it won’t work. The obstacle can be the group aspect and the patient group aspect. » (SP2)*
	Day and time	*« It’s true that a time slot during working hours also has its limits. Because when patients return to work part-time or full-time, therapeutic or otherwise, it’s certainly complicated to free up time from 10am to 12pm, for example. (…) So I think the choice of time slot is important. » (SP3)* *« I would really prefer it to be during school hours because I have my children who go to school. (…) And there, it was annoying because I couldn’t pick them up. (…) So when we’re off work, I admit I prefer it to be during school hours. »* (P8)
	Distance between home and the fencing gym	*« I wouldn’t have been willing to travel miles to participate in an activity. (…) I think it must be difficult to reach people who are further away to come and do an activity. That’s my point of view. »* (P9) *« I think the main obstacle was distance. It was the people who didn’t live nearby. (…) It’s true that some people gave up because of the distance. (…) On the other hand, people who live in Vandoeuvre or the greater Nancy area are very happy to find something nearby. » (SP2)*

### 3.2 Effectiveness

The effectiveness of the RIPOSTE pilot trial is presented in **Tables 4** and **5**.

**Table 4:** Effectiveness of early RIPOSTE versus delayed RIPOSTE on global health status.

			Difference of changes			
	Early RIPOSTE	Delayed RIPOSTE	Univariate		Multivariate	
	Mean (95% CI)	Mean (95% CI)	β [95% CI]	p	β[95% CI]	p
QLQ-C30 global health status						
**M0**	68.9 (17.1)	54.5 (23.2)	–		–	
**M3-M0**	+10.2 (-5.1 – 25.5)	+4.2 (-4.8 – 13.2)	+6.1 (-9.9 - 21.1)	0.43	+4.2 (-10.5 - 19.0)	0.57

**Table 5:** Evolution of scores (QLQ-C30, Global Shoulder Functional Capacity, QLQ-BR23, MFI-20, HADS) at M0, M3 and M6 in each group.

	Early RIPOSTE group			Delayed RIPOSTE group		
	M0	M3	M6	M0	M3	M6
	N=11	N=10	N=9	N=13	N=13	N=10
QLQ-C30						
Physical functioning	90.3 (12.06)	92.0 (11.11)	92.6 (12.67)	73.8 (20.45)	80.0 (16.63)	82.7 (18.91)
Role functioning	81.8 (30.23)	88.9 (23.57)	88.9 (23.57)	51.3 (35.66)	65.0 (40.41)	78.3 (27.27)
Cognitive functioning	86.4 (22.13)	92.6 (16.90)	94.4 (16.67)	70.5 (34.80)	81.7 (16.57)	78.3 (22.29)
Emotional functioning	75.0 (28.38)	78.7 (15.65)	82.4 (27.78)	63.5 (27.12)	79.2 (24.61)	79.2 (22.65)
Social functioning	90.9 (17.26)	88.9 (23.57)	88.9 (23.57)	61.5 (35.61)	86.7 (24.60)	83.3 (22.22)
Fatigue	27.3 (28.70)	21.0 (16.14)	16.0 (19.33)	48.7 (25.47)	54.4 (30.29)	43.3 (33.31)
Pain	24.2 (26.21)	22.2 (32.27)	16.7 (33.33)	42.3 (24.17)	35.0 (29.87)	28.3 (34.29)
Nausea and vomiting	1.5 (5.03)	5.6 (16.67)	3.7 (7.35)	1.3 (4.62)	11.7 (13.72)	5.0 (8.05)
Sleep disturbance	27.3 (38.92)	29.6 (35.14)	18.5 (33.79)	38.5 (35.61)	26.7 (26.29)	20.0 (23.31)
Constipation	12.1 (30.81)	18.5 (29.40)	0.0 (0.00)	10.3 (16.01)	16.7 (32.39)	16.7 (23.57)
Dyspnea	12.1 (22.47)	11.1 (16.67)	11.1 (16.67)	28.2 (26.69)	16.7 (23.57)	20.0 (32.20)
Diarrhea	3.0 (10.05)	0.0 (0.00)	14.8 (33.79)	15.4 (25.88)	10.0 (22.50)	13.3 (17.21)
Appetite loss	18.2 (34.52)	11.1 (33.33)	3.7 (11.11)	17.9 (17.30)	26.7 (37.84)	20.0 (28.11)
Financial difficulties	3.3 (10.54)	3.7 (11.11)	3.7 (11.11)	0.0 (0.00)	10.0 (31.62)	10.0 (22.50)
Global health status	68.9 (17.12)	74.1 (18.37)	73.1 (16.55)	54.5 (23.23)	61.7 (21.94)	66.7 (16.20)
Global shoulder functional capacity						
DASH score	17.9 (18.82)	13.0 (17.64)	9.3 (17.13)	38.4 (20.28)	27.2 (17.77)	21.0 (15.29)
QLQ-BR23						
Body image	68.2 (26.83)	68.5 (26.93)	63.0 (37.99)	48.3 (26.19)	57.2 (22.99)	59.2 (21.72)
Sexual functioning	28.8 (35.03)	31.5 (33.79)	31.5 (33.79)	19.4 (18.58)	25.0 (21.82)	27.8 (23.57)
Sexual enjoyment	66.7 (33.33)	58.3 (16.67)	66.7 (33.33)	33.3 (33.33)	53.3 (29.81)	60.0 (27.89)
Future perspective	48.5 (27.34)	51.9 (33.79)	58.3 (34.50)	46.2 (28.99)	50.0 (28.33)	66.7 (35.14)
Systemic therapeutic side effects	13.4 (19.84)	15.3 (16.80)	13.2 (21.40)	15.0 (10.97)	16.2 (12.54)	18.6 (20.13)
Breast symptom	27.3 (19.40)	27.8 (15.02)	18.5 (17.57)	33.3 (19.25)	37.5 (29.72)	16.7 (14.16)
Arm symptom	21.2 (23.55)	12.3 (21.83)	6.2 (9.80)	29.9 (20.48)	25.6 (25.69)	17.8 (19.03)
MFI-20						
General and physical fatigue	23.0 (6.81)	21.1 (6.15)	19.4 (7.55)	29.5 (6.53)	29.2 (6.56)	26.6 (8.75)
Mental fatigue	11.9 (6.71)	10.7 (3.43)	10.4 (5.94)	15.7 (4.85)	16.2 (5.09)	14.8 (4.76)
Reduced activity	7.8 (3.84)	6.9 (2.37)	6.2 (3.11)	9.0 (2.86)	7.6 (2.99)	6.0 (3.09)
Reduced motivation	2.5 (0.69)	3.6 (2.01)	3.3 (1.41)	4.5 (2.30)	3.7 (2.16)	4.6 (1.65)
HADS						
Anxiety	7.5 (4.97)	7.3 (4.39)	6.2 (4.21)	8.7 (2.42)	6.8 (2.53)	7.2 (3.82)
Depression	4.3 (3.77)	3.8 (2.95)	3.3 (3.16)	5.2 (3.79)	5.0 (2.94)	4.1 (2.69)
Global anxiety and depression	11.8 (7.04)	11.1 (6.11)	9.6 (6.75)	14.2 (4.93)	11.8 (5.27)	11.3 (6.00)

#### 3.2.1 Quality of Life

Global health status (GHS) score at inclusion (M0) was 68.9 (17.1) and 54.5 (23.2) respectively for the Early RIPOSTE and Delayed RIPOSTE groups (**Table 4**). At M3, GHS scores increased by +10.2 [-5.1; 25.5] and +4.2 [-4.8; +13.2] respectively in the Early RIPOSTE and Delayed RIPOSTE groups corresponding to a greater, but not statistically significant, increase of +6.0 [-9.9; +21.9] in favor of the Early RIPOSTE group. Multivariate analysis also highlights a trend for a larger increase of (+ 4.2 [-10.5 – 19.0], p = 0.57) of the quality of life score of the Early RIPOSTE group.

#### 3.2.2 Global shoulder functional capacity

At inclusion, the standardized DASH score was better in the Early RIPOSTE group than in the Delayed RIPOSTE group (17.9 versus 38.4). This score improved in both groups at M3 (13.0 versus 27.2) and at M6 (9.3 versus 21.0), suggesting an improvement of functional capacity (**Table 5**). Change between M0 and M3 was not statistically significant (-0.7 [-15.0 – 13.6], p=0.91) (**Supplementary Appendix 1**).

#### 3.2.3 Fatigue (MFI-20), anxiety and depression

At baseline, the general/physical fatigue score was 23.0 in the Early RIPOSTE group and 29.5 in the Delayed RIPOSTE group (**Table 5**). For the Early RIPOSTE group, this score decreased at M3 (21.1) and M6 (19.4), reflecting an improvement in fatigue status. Similarly, in the Delayed RIPOSTE group, there was a decrease at M3 (29.2) and M6 (26.6). In terms of mental fatigue, at inclusion, the score was better in the Early RIPOSTE group than in the Delayed RIPOSTE group (11.9 versus 15.7). At 3 months, this score improved in the Early RIPOSTE group but worsened in the Delayed RIPOSTE group (10.7 versus 16.2). At 6 months, this score improved in both groups (10.4 versus 14.8). Results were similar for anxiety and depression scales (**Table 5**).

#### 3.2.4 Physical activity

No difference between groups was observed regarding the level of physical activity.

### 3.3 Adoption

#### 3.3.1 Participation and withdrawal rates

The participation rate in the sessions was between 57% and 93%. One patient was lost to follow-up at 3 months and 4 at 6 months (refusal to continue). This corresponds to a withdrawal rate of 4.2% at 3 months and 16.6% at 6 months (**Appendix 2 Supplementary Data**).

#### 3.3.2 Barriers and facilitators to adoption

Barriers and facilitators to adoption are described in **Table 6**. The facilitators were the discovery of a new activity, good supervision of sessions and the physical and moral benefits associated with the practice. In contrast, participants cited contrasted laterality, fatigue and pain as the main barriers. In terms of organization, the majority of participants were satisfied with the duration (2 hours) and frequency of the sessions, although some would not have minded a second session a week, or regretted that the sessions were interrupted during school vacations. The size of the group seemed to suit everyone when it was reduced to around ten participants, with an even number to be able to work in pairs. While most participants appreciated the diversity and richness of the sessions, which were judged to be adapted to everyone’s abilities, some complained of boredom due to the lack of intensity. The facilitators were also the heterogeneity of profiles, and the cooperation and mutual support between participants, whatever their individual talent. However, integrating new participants during the program and reteaching them the basics seemed to disrupt and slow down the group’s progress somewhat. The good atmosphere and cohesion of the group, as well as the exchange and sharing of cancer-related experiences, also contributed to the adhesion of some participants, fostering a strong sense of community and belonging. Conversely, some saw the program as an opportunity to get away from the context of their illness and treatment.

**Table 6:** Barriers and facilitators to the adoption of the RIPOSTE.

	Category	Quotes
Facilitators	Discovery of a new sport	*« I liked learning fencing. (…) It is a very complete sport. (…) It plays on the physical aspect but also on the coordination of movements. Because there are many movements to coordinate at the same time. (…) It’s a very complete sport, I didn’t think so at all, but it allows you to work the whole body, legs as well as arms and head. »* (P5) *« I’ve had nothing but positive feedback, saying: “Well, it’s enabled us to discover this sport which we didn’t necessarily know existed within the framework of adapted PA (…) “. So, all in all, it’s been a great discovery. » (SP1)*
	Physical and moral benefits	*« We have a good time and it’s relaxing. We are relaxed, we work and it is extremely good. It’s good for the morale and for the body. I would like to praise the benefits it brings, both morally and physically. »* (P5) *« I found it very good, because in terms of movement, it allowed a certain amount of re-education. (…) It was good re-education for my chest and arm. »* (P7) *« None of them give up anyway. (…) They fight, they believe in it. (…) There’s no need to push them because they feel it’s for their own good. (…) That it’s good for their health, that’s a plus too. » (FM)*
	Duration of the sessions	*« At first, when I was told that the sessions lasted from 10am to 12pm, I thought I wouldn’t like it because I get bored quickly. But finally, with an hour of warm-up and an hour of activity, I don’t see the 2 hours passing. So I think it’s good. »* (P2)
	Frequency of the sessions	*« Once a week is enough. (…) More would be more complicated. (…) I think twice a week is a lot. »* (P2)
	Number of participants in the group	*« There are about ten of us and I think that’s good. More might be too much. But 10 to 12 participants is good, I think. »* (P5)
	Diversity and richness of fencing sessions	*« This is good because we never do the same warm-ups and exercises. (…) In fact, every session is different. That’s what I like, it’s not repetitive, we don’t do the same thing. »* (P2) *« I always (…) challenge myself to do something different, or try out different things. And you prepare a session and it may start out quite differently, as it often does. And then you do it again the next time, you try it again the next time. And often, people also bring a lot of things to the table, and (…) that gives you ideas for adapting. Adaptability and pedagogy are everything. » (FM)*
	Heterogeneity of partricipants’ PA levels	*« It’s good that we are totally heterogeneous groups with people who have never done sports, with people who do a lot, with people who have done other things. It’s good that we’re not all the same. » (P1)* *« Between the virulent, fast-moving people and the calmer, more composed ones … This sport allows them either to surpass themselves, or to slow down. And in the group, there are always people who are more or less active. (…) And that was interesting for them. » (FM)*
	Level of fencing sessions	*« It is really adapted to each one, that is to say really to our physical condition. The rhythm and the physical condition of each person are really respected. (…) We weren’t competing, we were really there to feel good about each other. Everyone does things at their own pace. »* (P3)
	Cooperation and mutual support between participants	*« The ones who started before or have more experience make the ones who have a little less experience work. » (P2)* *« By mixing the groups, it allows them to have more contact and take care of each other, because some are stronger or weaker than others. I felt that people helped each other. (…) It allowed us to go a little further in exchanges. (…) It was enriching for participants. » (FM)*
	Group atmosphere	*« I remember going to the weapons room to watch the sessions. (…) And to see patients who are dynamic, who want more, who smile, who are in a good mood. It’s like a breath of fresh air, in fact, and it does a lot of good. » (SP2)*
	Exchanges and share experiences about cancer	*« I became more of a fighter (…) when I started RIPOSTE. I felt better because I saw other ladies, other people and talked to them. (…) It’s a good thing because it allows you to connect with people and deal with your situation better. Because otherwise I was amplifying things a lot. And so, the fact talking with people allowed me to calm down. (…) I put things into perspective a little bit, that’s very important. »* (P7) *« As it happens, the people who go there most often are those who enjoy contact and have made friends. They’ve exchanged ideas on all sorts of subjects, they’ve experienced similar things, they have advice to give each other. So that’s one of the positive points on a social level. It’s also the fact of being with people who understand what they’re going through, because they’re going through the same thing. So there’s this aspect of sharing, of sharing the difficulty or the care pathway. » (SP2)* *« I feel they get something out of it in terms of sharing. It’s a group. It’s an individual sport, but it’s an individual sport practiced in a group. They know that it’s a suspended time, a time dedicated to them during which they can exchange with people who have had a similar life experience. And that (…) was good for them. » (SP3)*
	Supervision of sessions	*« Professional, a great listener, (…) you can see that he is someone who knows his job. (…) And he was concerned about listening to us, giving us all the leads so that we could give the best of ourselves. »* (P3) *« I found (…) that there was a lot of training for fencing masters. This is reassuring, because it’s true that we don’t want our patients, who are quite fragile, to end up in the hands of people who aren’t very careful. So there was something reassuring in the program’s thinking and in the training of fencing masters. » (SP2)* *« In any case, I had a very clear vision (…) of the character of the fencing master, who was also very important in the program. He’s a wonderful person, very caring. And that’s something I could easily tell my patients, and I had total confidence in sending them to him. » (SP2)*
Barriers	*Contrared laterality*	*« I don’t really like (…) sword sports. Maybe it’s because of the use of the left arm. I find myself less operational, less valiant, I find myself a little clumsy and maybe it’s because of that. (…) I don’t think I’m very good at making movements with that arm. I don’t think it’s for me. »* (P7)
	Fatigue and pain	*« Some didn’t come back anymore. (…) So he (the fencing master) told us that they had stopped. People who had joint pain, who didn’t feel up to it. (…) The goal was to not trigger more pain by doing 2 hours of rehab. »* (P10) *« Some people are still very, very, very, very tired. And even if it’s adapted, they find it hard to recover. » (SP3)*
	Frequency of the sessions	*« If there had been two sessions per week, I wouldn’t have minded. I find that from one week to the next, when you discover a sport, there is a time to acquire all the reflexes and all the vocabulary, it’s a bit special. So twice a week wouldn’t have been so bad for me. »* (P1)
	School vacations	*« There are no sessions during school vacations, and that’s another problem. (…) Because it’s true that we are sick all the time, even during school vacations! And we are available. In fact, it’s a bit frustrating that we have to stop. »* (P8) *« The other obstacle is the design of the program, which means that it doesn’t run during the vacations. And it’s true that in France, vacations come and go all the time. So there are a lot of interruptions, and it’s true that it’s very breakable. (…) I think that if it could be all year round, it might be even better. So school vacations are a hindrance for me. » (SP3)*
	Number of participants in the group	*« It depends on the week. Some weeks there were 8-10 participants, so it was fine, it was good. I remember one week there were 16 of us and it was getting to be a lot. Because some people would scatter and talk in the corner. (…) And as he (the fencing master) works with each one of us, it necessarily takes more time when there are 16 of us. So it’s true that ideally, I think a maximum of 10 would be good. »* (P1)
	Lack of exercise intensity	*« It is rather relaxed and friendly. (…) Sometimes it could be a little more dynamic, there could be more intensity. »* (P6)
	Arrival of new participants	*« The only small drawback was that new people arrived every week. So it was not easy to evolve because you had to start from scratch each time. (…) And I understand that it was not easy for the fencing master either because he had to adapt to each new person who arrived, to start again from the basics etc. (…) It’s a pity because it doesn’t allow you to evolve.»* (P10)

### 3.4 Implementation

#### 3.4.1 Consistency of implementation and adaptations

##### 3.4.1.1 Time and settings

The intervention was implemented according to the organizational context described in the protocol, with one fencing session per week for 3 months for each of the two groups. The Early RIPOSTE Group attended sessions on Monday at 4:00 p.m., while the Delayed RIPOSTE Group attended sessions on Friday at 10:00 a.m. The duration of 1-1.5h/session was deemed insufficient by participants and fencing masters, and was therefore extended to 2h. Sessions were sometimes interrupted due to school vacations or the absence of the fencing masters. These unforeseen events were well managed thanks to good communication within the group, especially via social networks. The number of participants fluctuated with each session and was not limited to 10-12 as planned. This was because some participants started the program later, were absent or dropped out.

##### 3.4.1.2 Structure of sessions

The sessions were divided into two periods of one hour each: a warm-up time dedicated to muscle awakening and progressive cardiorespiratory solicitation, followed by varied and adapted fencing exercises. The participants trained during lessons with the fencing master and among themselves. There were also regular breaks and exchange times according to the needs of the participants. When the number of participants was odd, they could not work in pairs. And when the number of participants was too high, the fencing master did not have enough time to work individually with each participant.

##### 3.4.1.3 Equipment and materials

The sessions included the use of sabers and dummies. In some sessions, fencing outfits with jackets and masks were loaned to participants. One participant complained that there were no outfits in her size, and another that the mask did not fit her hearing aids.

### 3.5 Maintenance

#### 3.5.1 Continuation of RIPOSTE or physical activity

Almost half of the participants planned to continue fencing, either in the RIPOSTE program or outside of it. Some wanted to continue in an adapted PA program, while others wanted to continue in a normal setting. For the other participants, the program made them aware of and motivated them to start new activities or to resume their previous activities. They felt more able and willing to pursue PA because of RIPOSTE but they wanted to move on to other types of PA, especially individual ones. Finally, a small minority of participants did not plan to continue PA, being mainly constrained by their schedule or physical condition.

#### 3.5.2 Prospects for change or improvement

According to sports physicians, it seems necessary to improve patient recruitment to optimize participation. Better communication about PA by all healthcare professionals involved in patient care could be a good solution in this respect. Sports physicians could also show fencing videos to their patients during consultations. Beforehand, healthcare professionals could be made more aware of and trained in the issue of PA and cancer, or even attend fencing sessions themselves, to help them get the message across to their patients. To improve patient follow-up and support, it may also be necessary to formalize exchanges between sports physicians and fencing masters. Other professionals involved in patient care, such as psychologists, could also be involved in this collaboration. Patient support could also be optimized at the end of the program, either through group discussion or individual follow-up consultations. The interviews also revealed prospects for change linked to the organizational context of the intervention. These changes concern the duration of the sessions, which could be set at 2 hours, and interruptions during school vacations. Participants also suggested that session times be better aligned with school and work schedules. The question also arose as to whether a second session per week, at different times, would be necessary to best accommodate participants. To overcome the obstacle of distance, we could consider having the fencing master travel to extend the program to the whole of the target area, or cover the participants’ transport costs. Regarding the size of the group, it seemed preferable to all that it be small (10–12) and stable, with an even number of participants. Finally, the interviews also highlighted the importance of considering participants’ particularities (e.g., vision or hearing problems) to ensure that the equipment is adapted.

## 4 Discussion

To achieve greater dissemination and generalization of PA programs, one of the current challenges is to address the lack of translation of evidence-based research protocols into practice (38, 39). A recent review by Klesges et al. highlighted the importance of considering dissemination when designing health promotion programs (40), but few behavioral intervention evaluations reported evidence of external validity of RE-AIM criteria (41). To our knowledge, our pilot study is the first to evaluate the RIPOSTE program effectiveness and implementation process using the RE-AIM framework. The RE-AIM model proved useful in highlighting the complexities of RIPOSTE implementation and evaluation, which could lead to a more complete program that addresses issues related to improving the external and internal validity of translating research into practical-generalizable practice.

Reach among all patients was good (24 patients, i.e. around a third of eligible patients). Most participants were young and dynamic and had expectations and beliefs about program outcomes. Similarly, BC survivors who exercise regularly report significantly more positive attitudes towards their physical condition, higher body esteem, better mood and higher vigor than sedentary (42). Psychological or physical, barriers to participation were mainly related to fatigue and pain. Difficulties in reaching older or sedentary patients could then be explained by differences in their existing abilities, but also in their motivation and beliefs. Previous studies have established that there are significant differences in perceived barriers to PA as a function of age and activity level (43). Older people’s motivation to engage in PA is particularly influenced by socio-cognitive variables such as their perceived physical frailty and poor health, perceived self-efficacy, perceived social support or perceived benefits/barriers to continued PA (44). It has been shown that older adults may have prevailing social beliefs that exercise is inappropriate, or even harmful, for them, or that they may be unaware of the importance of regular PA in preserving their health (45, 46). Participation may also be influenced by PA preferences, which are subjective constructs that prioritize choices, influenced by structural, interpersonal and intrapersonal factors (47, 48). The higher level of participation among young patients could be partly due to the match between the sports on offer - described as innovative and original - and their preferences (48, 49). Taking these preferences into account could make it possible to adapt the offer and support the involvement of all patients in PA. This highlights a further reach challenge in trying to recruit older, sedentary patients, as these are often the ones who need it most.

There is a high level of evidence supporting the benefits of PA programs in breast cancer. More precisely, PA interventions have been shown to enhance QoL, reduce symptoms and adverse effects of medical treatments and above all decrease the risk of recurrence and comorbidities (3–9). However, less information was available regarding the nature and the timing for such PA interventions in breast cancer. In a systematic review (50) aimed to assess the effects of Olympic combat sport on health QoL, only 6 studies were available and only one concerned fencing and breast cancer (51). Overall, it appears that interventions based on Olympic combat sports produce beneficial effects on health QoL. Another review also showed that Olympic combat sport improves older adults physiological and physical health (52). Our study (10) allows to more precisely explore the potential effects of adapted fencing after breast cancer surgery, considering a different timing among patients (i.e. “Early” vs “Delayed” RIPOSTE program). Therefore, we can’t conclude to a superiority (in terms of QoL) of starting RIPOSTE early (i.e. 4 weeks after surgery) vs starting later (i.e. 3 months after surgery). The lack of statistical significance of the results despite an overall trend towards a more favorable effect in the “Early RIPOSTE” group can however be explained by various reasons. First, the small size of our pilot study doesn’t allow to have enough statistical power to show a significant difference. This is consistent with the calculation of the minimum detectable difference when planning the study. Indeed, we had calculated a predicted minimum detectable difference of 12 points of QoL, but the observed difference is 6.1 points. This should be considered to adjust the hypotheses for calculating the number of subjects required when setting up a larger study in the future. In addition, we also observed differences in clinical characteristics and perceived health at baseline between our two groups (DASH and HADS scores). Indeed, almost all of the baseline clinical and perceived health criteria, were more favorable in the “Early RIPOSTE” group compared to the “Delayed RIPOSTE” group. Of course, probabilistic randomization was done, but it could not correct these initial differences. It would then be appropriate in a future study to increase the size of the included population also allowing to target or identify more particularly the patients according to diseases stages (subgroup analysis) to help highlight a difference.

On the other hand, and as a corollary to our results (i.e. only a trend for a better improvement of QoL in the “Early RIPOSTE” group), we can also suggest, because of the absence of significant difference between the 2 groups, that starting adapted fencing early is not more harmful than starting it later. This message is important, because it’s still regularly considered that a rapid/early resumption of physical activity can be harmful for the patient (risk of injury, risk for the surgical scar in particular). Offering an adapted fencing program, with trained fencing masters and under cover of medical examinations (performed by certified sports medical doctors) should therefore be considered as key elements of the RIPOSTE program, and also a guarantee of quality and patient’s safety. The qualitative analysis also provides interesting feedback on the RIPOSTE program, showing that it has been largely adopted and implemented.

Data demonstrate that participants adopted the program, largely because of the physical and moral benefits associated with the practice, regardless of the timing of the intervention. These beneficial effects were probably influenced by the diversity and richness of the exercises proposed, which were appreciated, as well as by the good group cohesion, support and exchanges that punctuated the sessions and facilitated the patients’ experience of the disease. In line with previous research, camaraderie and friendship within a small group are important reasons why BC survivors adopt PA (53, 54), helping them to feel accepted and supported by “similar others” (54). Some participants were motivated by sharing experiences around cancer, while others adopted the program because it enabled them to distance themselves from their illness. RIPOSTE offered women the opportunity to define and listen to their own specific needs and interests. The program thus seems to have fostered autonomy and behavioral control, key factors in behavior change according to the theoretical underpinnings of the self-determination theory (55) and theory of planned behavior (56). Financial barriers are one of the reasons why many BC survivors don’t make PA a priority after treatment (57). The free nature of the program has played a key role in its adoption by patients, offering them the opportunity to try an activity they hadn’t considered or didn’t think was aligned to their capabilities. We also feel it’s important to reconsider the organizational aspects that could act as a barrier to adoption, by adapting more closely to participants’ availability, particularly in terms of school or work schedules, and by remedying the interruption of sessions during school vacations. The lack of intensity mentioned by some participants as a barrier to adoption could be linked to the size of the group, which is sometimes too large, or to the arrival of new participants during the program, which slows down the group’s progress. Despite this, our results emphasize the success of the program, which depended in part on good supervision of the fencing masters. To ensure the sustainability of the intervention, it is essential to ensure that participants receive adequate supervision and support.

Implementation of the protocol elements was stable throughout the intervention. Assessing barriers to implementation while the program was ongoing enabled professionals to modify the organization and overcome the most common barriers, for example by setting the duration of sessions at 2 hours. Fencing masters also used social networks to communicate with participants and deal with unforeseen circumstances. In future, setting the number of participants at 10 or 12 would enable participants to train in pairs under optimum conditions, with the opportunity to work individually with the fencing master. Particular attention will need to be paid to the equipment, so that it is adapted to the patients’ physical particularities.

By creating a motivating and supportive environment, group PA programs are considered an effective way of encouraging BC survivors to maintain PA (54). As many participants started to notice positive changes in their health (e.g. arm and breast rehabilitation and reduced fatigue), it seems that their motivation to maintain PA after the program was enhanced, as indicated in the interviews. However, we have little follow-up data on this dimension, as participants’ continuation of PA has not been evaluated over the long term. The lack of reported long-term maintenance information is problematic, as it limits the estimation of the long-term impact of the program. We believe that additional strategies are needed to encourage and support the continuation of the intervention. Patient follow-up and support could be improved, for example by implementing collaboration and communication strategies between sports physicians and fencing masters, but also with other healthcare professionals such as psychologists. Another idea for doing so would be to add follow-up assessments, which would take place at least 6 months after the end of the intervention, and which would report on its degree of sustainability.

## 5 Conclusion

This randomized pilot trial mainly reached young and dynamic patients, and demonstrated strong effects in the dimensions of adoption and implementation. While organizational factors such as time, setting and equipment have a major influence on the successful adoption and implementation of this type of intervention, the implementation of follow-up and support strategies for patients seems essential to adapt the offer to their needs and preferences. Participants’ abilities, social support or any physical and/or psychological difficulties are all factors to be taken into account when offering APA in a context as specific as that of cancer. Intervention effectiveness and maintenance require further attention and innovation to confirm the overall positive trends. Increasing the size of the included population and the number of centers involved (i.e. multicentric study), and using more rigorous measures to assess PA maintenance are needed in future research.

## Data availability statement

The raw data supporting the conclusions of this article will be made available by the authors, without undue reservation.

## Ethics statement

The studies involving humans were approved by French ethics committee: Comité de Protection des Personnes Sud Méditerranée IV, N°ID-RCB: 2020-A01916-33. The studies were conducted in accordance with the local legislation and institutional requirements. The participants provided their written informed consent to participate in this study.

## Author contributions

SH: Writing – review & editing, Formal analysis. AV: Writing – review & editing, Writing – original draft, Validation, Supervision, Methodology, Formal analysis. MS: Writing – review & editing, Software, Methodology. CR: Writing – review & editing, Methodology, Funding acquisition, Formal analysis, Conceptualization. AC: Writing – review & editing, Project administration, Data curation. DP: Writing – review & editing, Validation, Supervision, Conceptualization. CD: Writing – review & editing, Supervision, Investigation, Formal analysis, Conceptualization. JR: Writing – review & editing, Supervision, Formal analysis. MT: Writing – review & editing, Supervision, Investigation, Formal analysis. EA: Writing – review & editing, Validation, Formal analysis. OH: Validation, Formal analysis, Writing – review & editing. BC: Writing – review & editing, Validation, Formal analysis. DH: Writing – review & editing, Validation, Supervision, Formal analysis, Conceptualization. AO: Writing – review & editing, Writing – original draft, Validation, Supervision, Project administration, Methodology, Funding acquisition, Formal analysis, Conceptualization. MP: Writing – original draft, Validation, Supervision, Project administration, Methodology, Investigation, Funding acquisition, Formal analysis, Conceptualization, Writing – review & editing.
