# Supplementary material for: Evaluating the feasibility and acceptability of an adapted fencing intervention in breast cancer surgery post-operative care: the RIPOSTE pilot randomized trial
Source: Front Oncol. 2024 Apr 11;14:1335442. doi: 10.3389/fonc.2024.1335442 (PMC11043494; doi:10.3389/fonc.2024.1335442)
Supplement: Supplementary file 1 [file DataSheet_1.docx]

Appendix 1: Effectiveness of early RIPOSTE versus delayed RIPOSTE on secondary outcomes

|  | **Change in “Early RIPOSTE”** | | **Change in “Delayed RIPOSTE”** | | **Difference of change** | | |
| --- | --- | --- | --- | --- | --- | --- | --- |
|  |  |  |  |  |  |  | |
|  |  | **Δ_1_=M3-M0** |  | **Δ_2_=M3-M0** |  | **β= Δ_2_ - Δ_1_** |  |
|  | **n** | **Mean (95%CI)** | **n** | **Mean (95%CI)** |  | **Mean (95% CI)** | **p** |
| **QLQ-C30** |  |  |  |  |  |  |  |
| Physical functioning | 9 | 3.1 (-8.0 - 14.3) | 10 | 6.0 (-5.6-17.6) |  | 2.9 (-12.1-17.8) | 0.69 |
| Role functioning | 9 | **11.1 (0.0 - 22.2)** | 10 | 13.3 (-12.9-39.6) |  | 2.2 (-25.4-29.8) | 0.86 |
| Cognitive functioning | 9 | 9.3 (-3.7 - 22.2) | 10 | 10.0 (-7.0-27.0) |  | 0.7 (-19.5-21.0) | 0.93 |
| Emotional functioning | 9 | 8.3 (-11.1 - 27.8) | 10 | **13.3 (3.1-23.5)** |  | 5.0 (-14.6-24.6) | 0.59 |
| Social functioning | 9 | 0.0 (-14.3 - 14.3) | 10 | 20.0 (-1.6-41.6) |  | 20.0 (-4.7-44.7) | 0.10 |
| Fatigue | 9 | -8.6 (-30.7 - 13.5) | 10 | 8.9 (-10.1-27.9) |  | 17.5 (-9.3-44.3) | 0.18 |
| Pain | 9 | -5.6 (-24.8 - 13.7) | 10 | -10.0 (-33.3-13.3) |  | -4.4 (-32.8-23.9) | 0.74 |
| Nausea and vomiting | 9 | 3.7 (-4.8 - 12.2) | 10 | 10.0 (-0.1-20.1) |  | 6.3 (-6.1-18.7) | 0.29 |
| Sleep disturbance | 9 | -3.7 (-27.5 - 20.1) | 10 | -3.3 (-31.9-25.2) |  | 0.4 (-34.5-35.2) | 0.98 |
| Constipation | 9 | 7.4 (-13.9 - 28.8) | 10 | 6.7 (-8.4-21.7) |  | -0.7 (-24.5-23.0) | 0.94 |
| Dyspnea | 9 | -3.7 (-27.5 - 20.1) | 10 | -13.3 (-25.6--1.0) |  | -9.6 (-33.5-14.3) | 0.40 |
| Diarrhea | 9 | -3.7 (-12.2 - 4.8) | 10 | -3.3 (-10.9-4.2) |  | 0.4 (-10.1-10.9) | 0.94 |
| Appetite loss | 9 | -11.1 (-29.2 - 7.0) | 10 | 10.0 (-15.3-35.3) |  | 21.1 (-8.3-50.5) | 0.14 |
| Financial difficulties | 8 | 0.0 (0.0 - 0.0) | 10 | 10.0 (-12.6-32.6) |  | 10.0 (-13.8-33.8) | 0.38 |
| **QLQ-BR23** |  |  |  |  |  |  |  |
| Body image | 9 | 3.7 (-7.0 - 14.4) | 10 | 0.3 (-19.9-20.5) |  | -3.4 (-25.4-18.5) | 0.74 |
| Sexual functioning | 9 | 9.3 (-5.2 - 23.7) | 8 | -2.1 (-19.5-15.3) |  | -11.3 (-31.8-9.1) | 0.25 |
| Sexual enjoyment | 3 | 0.0 (-82.8 - 82.8) | 3 | 33.3 (33.3-33.3) |  | 33.3 (-20.1-86.8) | 0.15 |
| Future perspectives | 9 | 7.4 (-9.7 - 24.5) | 10 | -0.0 (-15.9-15.9) |  | -7.4 (-28.9-14.1) | 0.47 |
| Systemic therapeutic side effects | 9 | -1.1 (-10.9 - 8.8) | 10 | 3.3 (-5.6-12.3) |  | 4.4 (-7.9-16.6) | 0.46 |
| Breast symptom | 9 | -2.8 (-20.0-14.5) | 10 | 3.3 (-8.3-15.0) |  | 6.1 (-12.7-24.9) | 0.50 |
| Arm symptom | 9 | -13.6 (-37.3-10.1) | 10 | -6.7 (-16.0-2.7) |  | 6.9 (-15.6-29.4) | 0.52 |
| **DASH** |  |  |  |  |  |  |  |
| Score | 9 | -8.5 (-19.1-2.2) | 10 | -9.2 (-20.2-1.9) |  | -0.7 (-15.0-13.6) | 0.91 |
| **HADS** |  |  |  |  |  |  |  |
| Anxiety | 9 | -0.8 (-3.6-2.0) | 9 | -1.6 (-3.6-0.5) |  | -0.8 (-4.0-2.4) | 0.61 |
| Depression | 9 | -1.1 (-3.4-1.2) | 10 | -0.1 (-1.5-1.3) |  | 1.0 (-1.4-3.4) | 0.39 |
| **MFI-20** |  |  |  |  |  |  |  |
| General and Physical Fatigue | 9 | -3.4 (-8.2-1.3) | 10 | 0.0 (-7.0-7.0) |  | 3.4 (-4.6-11.5) | 0.38 |
| Mental Fatigue | 9 | -2.1 (-7.2-3.0) | 10 | 1.2 (-3.0-5.4) |  | 3.3 (-2.7-9.3) | 0.26 |
| Reduced Activities | 9 | -1.8 (-3.8-0.2) | 10 | -1.2 (-3.7-1.3) |  | 0.6 (-2.4-3.6) | 0.69 |
| Reduced Motivation | 9 | 1.1 (-0.5-2.7) | 10 | 0.0 (-0.7-0.7) |  | -1.1 (-2.7-0.4) | 0.14 |

**Apperndix 2: Participation to the RIPOSTE sessions**

| **N°** | **Group** | **Loss to follow-up** | **Number of participated sessions** | **Total number of sessions proposed** | **Participation rate** |
| --- | --- | --- | --- | --- | --- |
| 1 | Delayed RIPOSTE | No | 14 | 15 | 93% |
| 2 | Early RIPOSTE | No | 13 | 15 | 87% |
| 3 | Early RIPOSTE | No | 8 | 12 | 67% |
| 4 | Delayed RIPOSTE | No | 12 | 15 | 80% |
| 5 | Early RIPOSTE | Yes | 3 and then withdraw |  |  |
| 6 | Delayed RIPOSTE | No | 10 | 12 | 83% |
| 7 | Early RIPOSTE | No | 8 | 14 | 57% |
| 8 | Delayed RIPOSTE | No | 10 | 12 | 83% |
| 9 | Delayed RIPOSTE | No | 9 | 12 | 75% |
| 10 | Early RIPOSTE | Yes | 1 and then withdraw |  |  |
| 11 | Early RIPOSTE | No | 12 | 13 | 92% |
| 12 | Delayed RIPOSTE | No | 10 | 12 | 83% |
| 13 | Delayed RIPOSTE | No | 7 | 12 | 58% |
| 14 | Delayed RIPOSTE | Yes | 2 and then withdraw |  |  |
| 15 | Early RIPOSTE | No | 9 | 13 | 69% |
| 16 | Early RIPOSTE | No | 10 | 13 | 77% |
| 17 | Delayed RIPOSTE | Yes | 3 and then withdraw |  |  |
| 18 | Delayed RIPOSTE | No | 10 | 12 | 83% |
| 19 | Early RIPOSTE | No | 10 | 12 | 83% |
| 20 | Early RIPOSTE | No | 13 | 14 | 93% |
| 21 | Delayed RIPOSTE | Yes | 2 and then withdraw |  |  |
| 22 | Delayed RIPOSTE | No | 10 | 12 | 83% |
| 23 | Early RIPOSTE | No | 13 | 14 | 93% |
| 24 | Delayed RIPOSTE | No | 10 | 12 | 83% |

**Appendix 3: Consistency of program implementation and adaptations**

| Category | Quote |
| --- | --- |
| Time and settings | *« An hour and a half may have been a bit short. (…) Now, at least, we have the time. (…) Some people come a little bit later, or leave a little bit earlier because they have constraints. So the amplitude allows everyone to come and work enough. (…) And it goes well and you don't see the time go by, so 2 hours, I found it good. » (P1)* |
|  | *« With the follow-up we put in place, (…) we went from groups of 7-8 to 23 participants. (...) It was really complicated. I slightly increased the passage time, which was originally 1h30. I increased it to 2 hours to have more rest time, since there were more people, and then to have individualized follow-up at each session, in several small steps, even if it's only two minutes each. » (FM)* |
|  | *« And now, with the phone, we are quickly informed when a session is cancelled or postponed. (…) With WhatsApp groups, when there was a time change, we knew. Or when there was a problem, at least we didn't go to the gym for nothing. » (P8)* |
|  | *« I simply asked them to let me know (...) and they played the game very well (...) It's important for the group to know why the person didn't come, because they had this or that problem, or were ill. Everyone in the group knew who was there, who wasn't and why. That's important too, and part of the value of the group. » (FM)* |
|  | *« The size of the group is never the same. One week you have 12 or 13 people. The next time, you have only half of them. (…) So it's never the same. » (P7)* |
| Structure of sessions | *« The exercises seemed complete. We worked on the legs, the shoulders, we worked a little on breathing. (…) And then there were the fencing exercises. We train with other people, and then at some point the fencing master takes us individually to correct us or to teach us something else. »* |
|  | *« It's very important to have individualized follow-up at each session (...) so that you can get back in touch with yourself, your illness and your suffering. (...) And that's what I tried to do every time. And then afterwards, there were times when they shared their knowledge, in the form of games or other activities. Breaks are also important, because everyone needs to be listened to and talked to. That's also part of the therapy, it's important. » (FM)* |
| Equipment and materials | *« The only complaint I would have is that there was no outfit in my size. (…) We were training with the fencing jacket and there was none in my size! » (P2)* |
|  | *« Wearing the fencing mask was a real horror for me. I came out of my session like a pressure cooker. (…) I couldn't hear anything because my ears were plugged (…) It was not adapted. When the mask pressed on my hearing aids, I heard whistling noises. (…) I had to tell him that I couldn't hear what he was saying, but he couldn't do anything about it.. » (P9)* |

Consistency of program implementation and adaptations

**Appendix 4: Maintenance of the program**

| Category | | Quotes |
| --- | --- | --- |
| Continuation of RIPOSTE or Physical Activity | Continuation of fencing | *« I will continue RIPOSTE until the end of the year. (…) And then I think I'll continue fencing because I find it very complete and I like it. So I think I will continue a little bit. » (P5)*  *« Every year, there are always 2 or 3 who continue. They don't fence for the rest of their lives. But there are always 2 or 3 who join and stay with the club for at least 2 or 3 years. Then we lose track of them, but that's not a problem, because they've had a slice of fencing life for 3-4 years, which is not uninteresting for them. » (SP3)* |
|  | Continuation of PA | *« I used to be very angry because I would get out of breath quickly and it would weigh me down. Doing fencing made me realize that these things were getting a little bit better with each session. (…) I have seen an improvement. At least, that's what made me decide to join a gym to do a workout at least once a week! That's it! And in September, I'm going to start doing water aerobics again. » (P3)*  *« On the whole, I've had good feedback, with some patients even wanting to continue afterwards. Others have opened their minds to physical activity and want to continue doing it, not necessarily fencing, but other activities afterwards. So the feedback has been pretty good. » (SP1)* |
|  | Non-continuation | *« I can't go back to sports now. (…) And I won't go back to sports in September either. I think it will take a while. (…) I don't have enough strength in my left arm. (…) In the meantime, I'll do something else quietly. » (P8)* |
| Prospects for change or improvement | Patient recruitment | *« In terms of areas for improvement, is it necessary to further improve communication with patients? (…) It's important, especially for surgeons who are the main providers of patients. I could also pass the message on to psychologists and nurse coordinators, who can also do a lot of recruiting. But yes, one area for improvement is better recruitment. » (SP2)*  *« If, at the end of a surgical consultation, the surgeon (…) can give even the most basic advice. (…) And if the message is then taken up by the oncologist, radiotherapist, sports doctor or anaesthetist, well, that's already a step in the right direction. So I think even the surgeon could be heavily involved in giving a minimum of advice, and dispelling sometimes erroneous ideas. (…) And the program will be all the more effective if all the players in the care chain are involved. » (SP3)*  *« In the first step, maybe show some explanatory videos, that's always pretty informative. » (SP1)* |
|  | Raising awareness among healthcare professionals | *« We're already doing it, but not enough, and we need to reinforce the message, in terms of pedagogy, teaching and continuing education. Or, at congresses, we could organize sessions on the benefits of physical activity for chronic diseases, and even more so for cancers. I think there's still a long way to go. » (SP3)*  *« If you really want to get people on board and trigger prescriptions, you need to know a minimum of what you're prescribing. This doesn't necessarily mean going into a fencing room, but the fencing master can also come into a hospital and show a little of what he does. Promote during major BC events. » (SP3)*  *« Of course, it would be great if the whole ICL breast group could come and meet the fencing master and have a fencing session to try it out. Surgeons could handle a sword and see what it feels like. Oncologists and radiotherapists could do it too. In absolute terms, on paper, I think it's very good. » (SP2)* |
|  | Collaboration between professionals | *« A good understanding between the prescribing doctor and the fencing master who delivers it. We need to know each other well. If the fencing master has a patient who no longer comes, he tells me. I think this is a real lever that should be in place everywhere, and isn't. (…) I think it's important to have fairly fluid communication between the person dispensing the physical activity, i.e. the fencing master, and his prescriber. This type of exchange (…) is not at all formalized. It's a question of knowing how to behave, but it's true that a dedicated time should perhaps be formalized. Regular, formalized milestones between the fencing master and the prescriber. (…) I think that as they stand today, they are insufficient because they are not formalized. (…) In fact, we could say that every 3 or 4 months, physicians and fencing masters could have a little progress report of one kind or another. Video-call, face-to-face, in the fencing room or hospital, it doesn't matter. The format doesn't matter, but taking an hour to get feedback from each other would be interesting, I think. » (SP3)*  *« We don't have much information about the other care they receive. For example, it's important to know whether patients receive psychological follow-up. And I don't really have a link with the psychologists who follow them. Maybe we need a bit more feedback from others. (…) That's still important and plays a part in adhesion, but we don't really have any feedback on that. (…) We could, for example, make a video-call at some point in the program and exchange information. Maybe that wasn't enough. Make a video-call at least once with the different healthcare players to see what everyone thinks, halfway through the program, before it's over. » (SP1)* |
|  | Patient support | *« I think patients should be seen at the end of the program so that it doesn't come to an abrupt end. There should be a follow-up consultation on adapted physical activity. You've done this program, you've enjoyed it, it's coming to an end, but that doesn't mean you should stop physical activity, and we can suggest that you continue, either by following up on it, or by looking at other options. But you shouldn't give up on them straight away at the end of the protocol, and you should also support them afterwards. » (SP1)*  *« Do the same thing as a group with patients (…) along the way or at the end of the year. (…) Take a dedicated time that's not an adapted fencing session, but a time for collective exchange so that everyone can take a look back at everything. And to make sure that everything is going well for everyone, quite simply. » (SP3)*  *« I know I always had little questionnaires at the end of the session (...) because I liked to get anonymous feedback from people. And then a little evaluation to see if everyone was satisfied overall, both on the sporting aspect and the social aspect (...) rather at the end of the cycle. (...) It was just to give an overall picture of the year as a whole. It's important to evaluate ourselves in relation to people's feedback. » (FM)* |
|  | Organizational context of the intervention | *« That would allow for two sessions a week. (...) Those who want to do more, they would do more. And the one who doesn't want to, she'd only come once. And the one who is not free on Friday would come on Tuesday or Monday. (...) This would give us a little more flexibility and allow us to progress a little faster. » (P1)*  *« Maybe multiply the time slots. Maybe offer a daytime slot, then an evening slot. (…) At the moment, there's one slot a week, but that's not much. Offer patients an alternative, with one or two slots. » (SP3)*  *« We could try to free ourselves from these constraints (…) by making the fencing master fly. (…) Relocating the fencing master to get away from geography could be a lever. I know we do it for schools, for children: they go to school, college, elementary school. So why not consider it under the right conditions, of course. » (SP3)*  *« It's the eternal question: if we can't have sufficient regional coverage, couldn't transportation costs be covered to some extent, maybe not completely, but in part? I don't know, but some form of reimbursement. We don't need an ambulance and a transport voucher either, but some kind of package or something so they can at least reimburse themselves for gas. I don't know, but it should be as neutral as possible for them. Because I think it's an obstacle anyway. » (SP3)* |
|  | Equipment | *« In terms of equipment, there's everything you need, but it may have to be renewed from time to time. There's also a life that goes on, and you have to buy from time to time to amortize the equipment and keep it in perfect working order. » (FM)* |

Maintenance of the program
